# Summer pruning in Mediterranean vineyards: is climate change affecting its perception, modalities, and effects?

**DOI:** 10.3389/fpls.2023.1227628

**Published:** 2023-07-11

**Authors:** Stefano Poni, Tommaso Frioni, Matteo Gatti

**Affiliations:** Department of Sustainable Crop Production, Universitá Cattolica del Sacro Cuore, Via Emilia Parmense, Piacenza, Italy

**Keywords:** vegetative growth, yield, grape composition, mechanization, source-to-sink balance

## Abstract

Summer pruning encompasses a series of operations typically performed on the grapevine during the growing season. This review provides an update on the research conducted over the last 20 years on the modalities and strategies of main summer pruning operations, which include shoot positioning and thinning, shoot trimming, leaf removal, and cluster thinning, with a special focus on their adaptation to climate change occurring in Mediterranean areas. Three main novelties emerged from the survey. First, due to a common need to shelter clusters against overheating and sunburn-related damages, shoot thinning and leaf removal are practices that are now being applied in a much more cautious and conservative manner. Second, the meaning of summer pruning is evolving because operations are being used as precious tools to direct ripening toward a desired direction rather than being received passively. Third, some operations, such as leaf removal, have disclosed very high plasticity, which means that, depending on the timing and modalities of the intervention, yield can be either increased or decreased and ripening anticipated or postponed. In an era where economic and environmental sustainability have to find a good compromise, cluster thinning is increasingly being depicted as an extraordinary operation that should be left to occasional occurrences of overcropping. Moreover, summer pruning is a tool through which growers can, to an extent, exploit the potentialities offered by climate change. For instance, the crop-forcing technique, under the different configurations of single and double cropping within the same season, has been trialed promisingly in several regions and cultivars. The principle of forcing is to unlock the dormant bud during the first year by removing at least the young organs present on the shoot within a time window between the end of the flowering and pea-size stages. In particular, when it is applied in a double-cropping mode, the preliminary results related to Pinot noir, Grenache, Tempranillo, and Maturana tinta indicate that two harvests separated by 30–50 days can be obtained, with the latter having superior quality in terms of a lower level of pH and higher levels of acidity, anthocyanins, and phenolics.

## 1 Introduction

Summer or green pruning encompasses any manual or mechanical operation performed in vineyards when even a minimal sign of vegetative growth is perceivable in the canopy. Therefore, in the strictest sense, even a very delayed winter pruning (Poni et al., 2022), performed, for instance, when buds on the canopy have already developed a few unfolded leaves, can be categorized as summer (green) pruning. The four main categories of summer pruning can be distinguished: shoot thinning and positioning, shoot trimming, leaf removal, and cluster thinning. All these share a common feature, i.e., to trigger a dynamic (and sometimes difficult to describe or quantify) seasonal change of the canopy leaf-area-to-yield (LA/Y) ratio, with possible impacts on the speed of ripening and based on the degree a given task changes the cluster microclimate. This feature renders the idea that any strategy for summer pruning operation unavoidably interacts with the ongoing and predicted climate change effects.

Among the examples that could be given to underline the aforementioned interaction, two are particularly effective. First, warming effects bound to climate change translate into a higher number of hot days as well as a higher frequency and/or severity of meteorological drought (Fraga et al., 2012; Schultze and Sabbatini, 2019; Van Leeuwen et al., 2019), leading to reduced canopy vigor. This explains why damages due to organ overheating, sunburn, and desiccation are nowadays considered increasing threats in vineyards. It is quite well established that several summer pruning operations do directly (i.e., leaf removal) or indirectly (i.e., a shoot trimming shifting the lateral regrowth on the apical part of the shoot and, consequently, limiting the cluster leaf cover from the basally located laterals) alter the cluster microclimate; therefore, we must question whether the technique of summer pruning needs to be adapted or changed. Second, it is similarly very stimulating that summer pruning could potentially be leveraged to address increasing complaints about environments with excessively fast sugar accumulation that becomes increasingly decoupled from phenolic and flavor ripeness.

If one perception is that the modalities of summer pruning need revisiting, perhaps a deeper change is at hand. Thanks to the vast knowledge that is presently available regarding the physiological background and expected effects of summer pruning operations, they can be turned into precious tools that the grower might utilize to orientate growth and ripening toward a desired direction, rather than perceive them as preemptive and unavoidable “things to do.”

The purpose of this review was to summarize the most recent findings about the most common summer pruning operations and determine whether there is any room to re-think their interventional modalities under the pressure of past, ongoing, and predicted effects of climate change having Mediterranean areas as the main focus.

## 2 Shoot positioning and thinning

**Figure 1:**
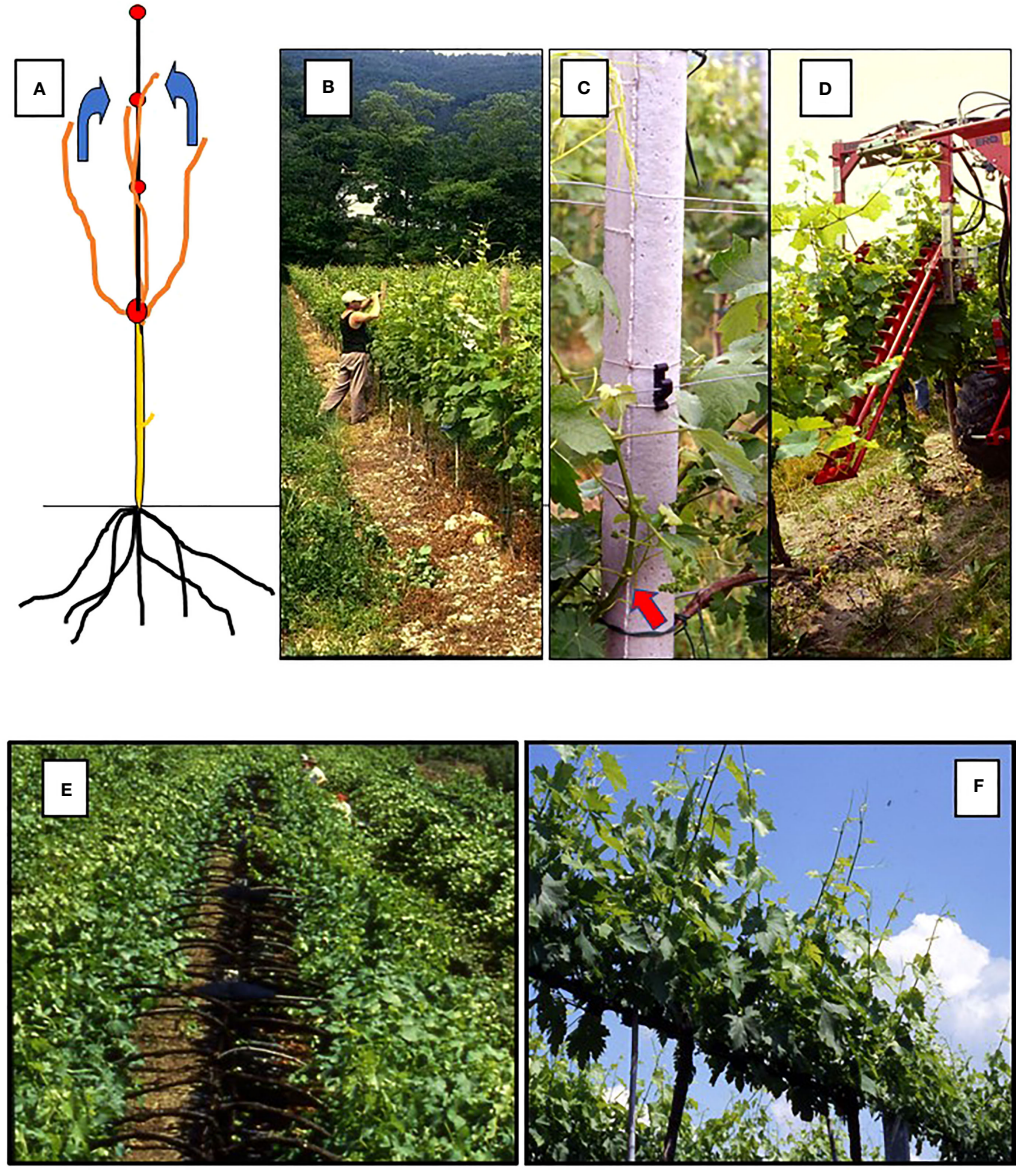
Diagram of a vertical shoot positioning (VSP) training system **(A)** and of hand **(B)**, plastic-hook aided **(C)** and mechanical **(D)** shoot positioning, The red arrow in panel B indicates the basal shoot nodes from which next season spurs will be obtained. Bottom panels: in **(E)** manual shoot positioning under way in a Geneva Double Curtain (GDC) training system: it is apparent how the operation is crucial for maintaining the two parallel canopies physically separated; in **(F)** a sprawl canopy type (single high wire cordon) which does not need any shoot positioning.

In the context of vineyards, shoot positioning has recently acquired a broader meaning, depending upon the evolution of training systems. In fact, at present, traditional shoot positioning, which pertains to vertical shoot-positioning (VSP) hedgerow trellises (**Figures 1A–D**), has a few alternatives linked to a given training system. For instance, in the case of Geneva Double Curtain (GDC) system, positioning refers to as the action of moving the shoots growing inward to an outward position (**Figure 1E**). This is because the two “divided” curtains will have to maintain a spatial separation during the season while radiation is allowed to reach the inner part of the two curtains. Then, depending on the choice of trellis (e.g., goblet or single high-wire cordon, with both featuring no foliage wires for shoot attachment), shoot positioning might not be required (**Figure 1F**). In fact, in the case of a sprawling canopy, the main aim is to obtain a naturally and mostly erect canopy that, once assisted by moderate shoot trimming, does not need any other adjustment. The research conducted on Shiraz and Grenache trained for both VSP and sprawl types (Louarn et al., 2008) has shown that non-positioned shoot systems offer the possibility of combining a high level of light interception, proper sun exposure and ventilation around clusters, and reduced labor-intensive practices for vineyards under conditions of moderate vigor. (Zahavi et al., 2001) performed the same comparison on Chardonnay and Cabernet Sauvignon, which are grown in the Golan region of Israel. This led to the conclusion that the high level of irradiance assured by the sprawl canopies was effective at reducing powdery mildew infection in moderate- and high-level diseases.

From a strictly physiological perspective, it has also been shown that when sprawl-trained Chardonnay canopies were enclosed in plastic chambers to monitor daily and seasonal whole-canopy gas exchanges, and the same canopies were squeezed between catch wires to simulate a VSP pattern, the whole-canopy net CO_2_ exchange rate diminished by 26% (Intrieri et al., 1997). This demonstrates that the foliage packing typical of a VSP-constricted canopy impairs the efficiency of light utilization. Based on these positive outcomes that were once linked to the challenges imposed by the climate, it is apparent that the no-positioning option offered by the sprawl canopy type is a direction that should be pursued. This is not only useful in saving time during a notoriously labor-intensive operation but the dim light regime broken by sun flecks—which an open, semi-erect canopy can provide to the subtending clusters—is very likely a winning option when overheating and sunburn have to be mitigated (Mabrouk and Sinoquet, 1998; Downey et al., 2006; Tarara et al., 2008; Poni et al., 2018).

Shoot positioning is applied in a VSP trellis with the primary goals of avoiding shoots growing towards the alleyway, allowing better light exposure of basal nodes to be retained in winter for next year’s cropping (i.e., the case of short pruning) (**Figure 1B**), and, above all, building an ordered and uniform vertical canopy that can be ideally shoot-trimmed to the required number of main leaves. However, it is detrimental when a canopy with large gaps and/or several shoots invading the alleyways originates due to poor or absent shoot positioning, which is sometimes coupled with inexperienced wire distancing along the canopy wall (e.g., a situation where the first couple of catch wires are too distant from the main support wire, so most growing shoots deviate too soon from verticality and escape from the wire capture track). With mechanical trimming, these will be shortened to just a few leaves above the clusters, and their ripening potential will be compromised.

In certain viticultural regions, and especially in Tuscany, Italy, traditional shoot positioning followed by trimming in a VSP trellis is replaced with horizontally wrapping the growing shoots along the top wire and leaving them untrimmed. Faralli et al. (2022) in their two-year-long study on Cabernet Franc/SO4 grown in northern Italy, provided a direct comparison between the two solutions. They found that the wrapping treatment produced the highest polyphenol and anthocyanin contents as well as the highest must acidity. Two main reasons were provided to explain this effect: (i) higher shading is cast on clusters from more leaves concentrated at the top of the canopy and (ii) most of the laterals are still concentrated in the basal nodes as the wrapped shoots are mostly untrimmed and the apical dominance is left undisturbed, which contributes to the casting of additional leaf cover onto the clusters. When shoot wrap was applied on Cabernet Franc grown in the cool Finger Lakes Region (NY, US) no effects on grape quality were seen (Logan et al., 2021). However, in a climate quite conducive to cluster rot, shoot wrap also achieved the desirable features of reduced fruit zone lateral lengths by up to 50% and cluster compactness by up to 2.4 fewer berries per centimeter rachis.

**Figure 2:**
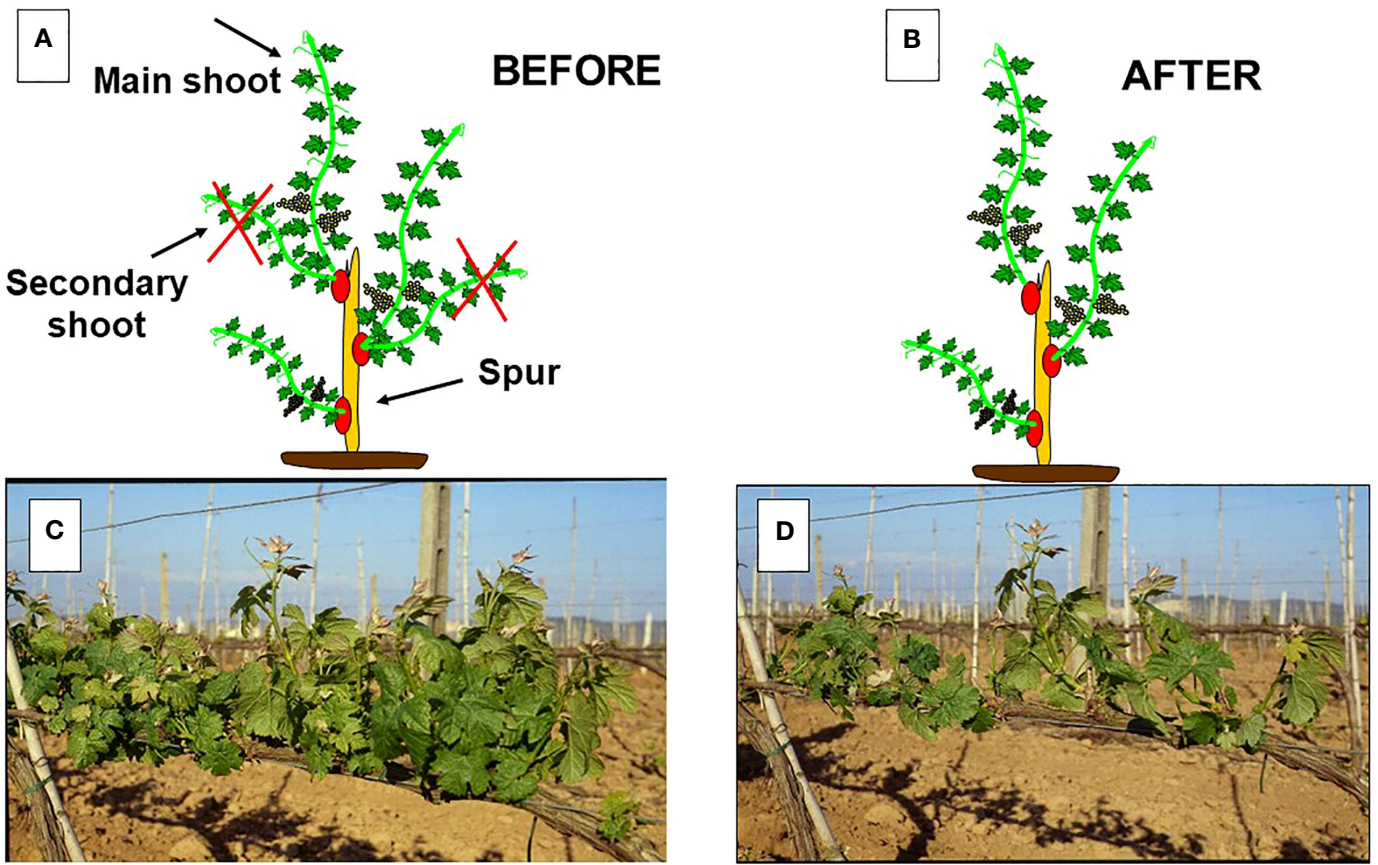
In **(A)**: representation of a spur with three count nodes which has originated a total of five shoots. In **(B)**: final set-up after manual shoot thinning which has removed the secondary shoots. In **(C)**: pre-thinning canopy density; in **(D)**: post thinning canopy density.

Shoot thinning is usually applied in viticulture in medium-to-high-vigor areas as a tool to prevent excessive canopy density at the cluster level targeting a final shoot density of around 10–15 shoots/m (Smart, 1985; Reynolds et al., 2005) (**Figure 2**). This situation occurs more often in cane-pruned systems when several double shoots are burst at each node, as well as in spurred systems when the development of primary shoots occurs with the concurrent growth of other shoots from secondary or base buds, thereby creating leaf clumping around the spur itself. Although the effects of shoot thinning have been somewhat neglected by the scientific community compared to those of other summer pruning operations, here is a summary of results obtained thus far: (i) as a result of an operation performed early in the season (the shoot length is, on average, around 15–20 cm), the growth compensation due to the remaining shoots usually allows for full recovery in terms of the final leaf area (Reynolds et al., 1994; Bernizzoni et al., 2011) or the total pruning weight per vine (Naor et al., 2002); (ii) a shoot thinning performed on Barbera vines, which reduced density from 30 to 15 shoots per meter, led to full canopy photosynthesis recovery at only 17 days after the thinning was performed (Bernizzoni et al., 2011); and (iii) in the large majority of cases, total soluble solids (TSS) and total anthocyanins increased with decreasing shoot density (Reynolds, 1989; Mota et al., 2010; Bernizzoni et al., 2011; Silvestroni et al., 2016) as result of reduced Ravaz index (i.e., yield-to-pruning-weight ratio) or increased LA/Y ratio (m^2^/kg).

As in the case of shoot positioning, it is important to question whether the objectives and effects of a shoot thinning operation are sensitive to climate change. A study conducted on Cabernet Sauvignon grown in Oakville (CA), focused on the effects of shoot thinning and leaf removal on flavonoid content, confirming the enhancing effect of shoot thinning on sugars, phenolic substances, hue, and proanthocyanins polymerization (Torres et al., 2021). However, it was also surprisingly found that shoot thinning decreased wine’s antioxidant capacity due to diminished catechin and quercetin content.

At present, when shoot thinning has to be performed to balance an otherwise excessively dense canopy, a limiting factor is that despite the deserving yet occasional attempts of mechanization (Kurtural and Fidelibus, 2021), the operation is manually performed and requires approximately 20–40 hours/hectare. This is mostly due to the difficulty in accessing the organs to be thinned and the high degree of selectivity required. However, today, robotics associated with the approaches of machine learning and artificial intelligence is seriously tackling the automation of this kind of operation, along with winter pruning (Guadagna et al., 2023; Teng et al., 2023). A similar approach is being taken for shoot thinning, with some preliminary research aiming to assess cordon shapes by using deep learning networks (Majeed et al., 2021). The preliminary results are quite encouraging. A sixth-degree polynomial model could fit approximately 80% of cordon trajectories with an R^2 ^= 0.98. Thus, this model might allow for the detection of cordons even if it is too heavily occluded by shoots to precisely position and orient thinning end-effectors for automated shoot thinning.

Considering the above-cited results together, it can be concluded that, when performed in areas allowing for good vegetative vigor, decreased shoot density leads to early/enhanced ripening, primarily because of an LA/Y ratio that might increase in quantity (especially when shoot thinning also regards some fruitful shoots, thereby lowering the yield level) and quality (especially when the growth compensation of the remaining shoots prolongs the formation of new leaves, which might then reach maturity at the right time to boost ripening) compared to non-thinned vines. Overall, when shoot thinning is used in warm environments where an excessively fast sugar accumulation often decoupled from adequate phenolic maturity is almost the rule, its use should be more cautiously regarded.

## 3 Shoot trimming

**Figure 3:**
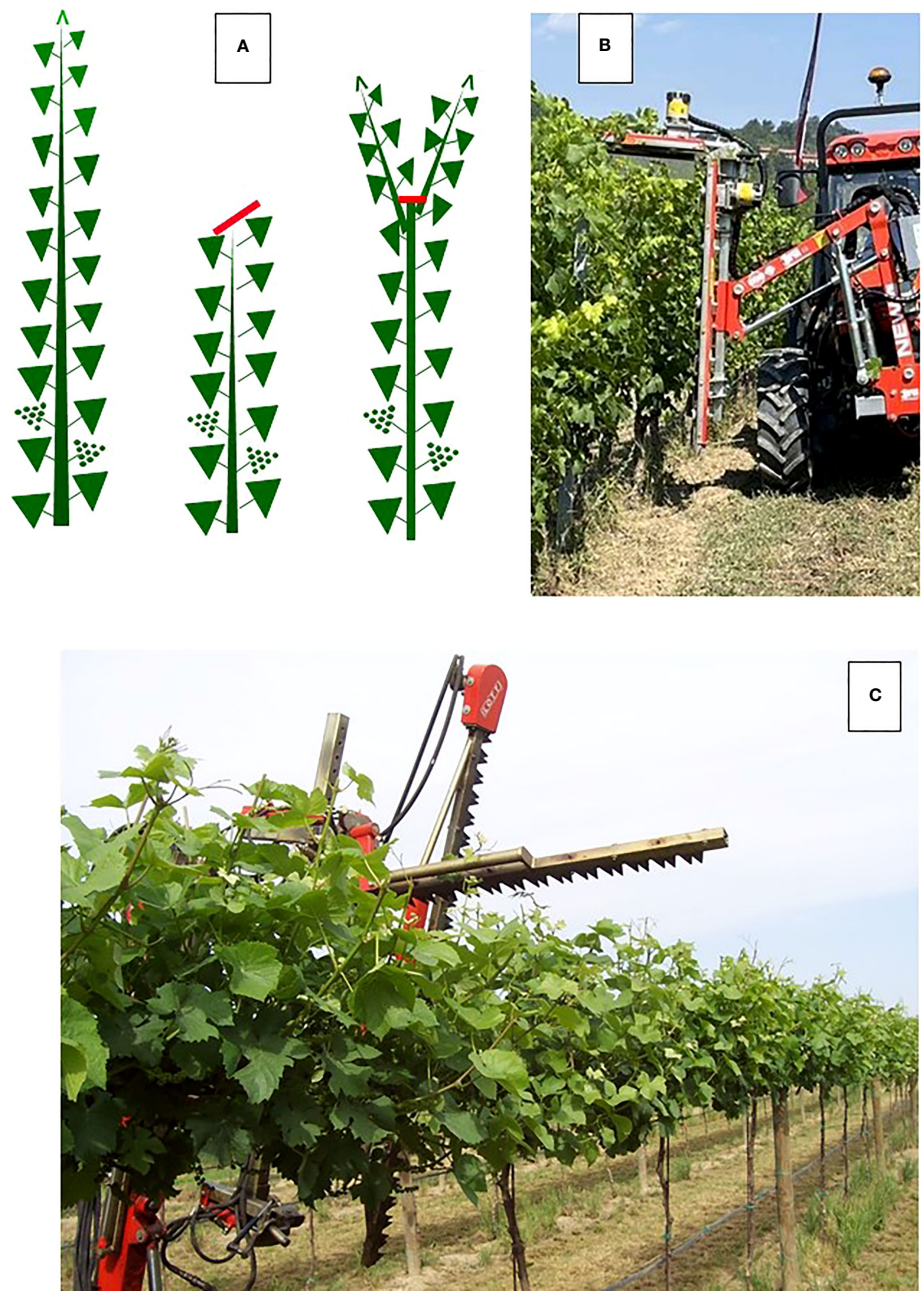
In **(A)** representation of a standard shoot trimming followed by the regrowth of some laterals. In **(B)** a cutter bar machine operating hedging and topping cuts in a vertically shoot positioning (VSP) trained vine row; in **(C)** the same machine at work on a sprawl, single high wire cordon. In the latter, cutter bars can operate even at a very close distance from the cordon as no foliage wires are present.

Among summer pruning operations, shoot trimming, which involves removing the shoot apex and some of the subtending young leaves (**Figure 3A**), is likely the best example of a practice that has been traditionally regarded as either neutral or mild in terms of affecting the yield and grape composition but is driven by the need of maintaining a more regular canopy size so as to not hinder the vineyard traffic and other vineyard operations (e.g., spraying or soil management). With more attention put into the physiological effects triggered by shoot trimming, this operation has been re-evaluated in terms of its ability to alter ripening dynamic, which might end up being either promoted or delayed depending on the timing and severity of the trimming, as well as the environmental conditions and the crop load after the trimming (Keller et al., 1999; Poni and Giachino, 2000; Mota et al., 2010; Dokoozlian, 2012; King et al., 2012; Poni et al., 2014b).

Today, in the context of climate change, the significance of shoot trimming has grown because it appears to be the right tool to obtain two concurrent advantages: on one side, reducing canopy size through shoot trimming would also result in lower canopy water use, whereas on the other side, if shoot trimming causes a permanent reduction in the LA/Y ratio, the ripening process could, consequently, be delayed.

With regard to former desirable effect, (Williams et al., 2022) confirmed the reliability of estimating seasonal grapevine crop coefficients (K_c_) from the shaded areas beneath grapevine canopies that, in turn, are a function of several factors, among which trellis geometry and canopy size changes induced by summer pruning are prioritized. However, although it has been demonstrated that adopting a split canopy such as the Lyra system leads to a mid-season K_c_ of 0.96 vs. 0.49 measured in a VSP trellis at the same between-row spacing (2.74 m) (Williams et al., 2022), the same proportionality is more dubious when the water use or water status of a tall, untrimmed canopy is compared to that of a canopy shortened by trimming. Over a three-year period, (Abad et al., 2019), conducted a study comparing the vine water status—assessed as stem water potential (Ψ_ST_)—of untrimmed and severely trimmed vines at berry pea size in different locations of northern Spain. Their findings revealed occasional and inconsistent differences in Ψ_ST_ between the two treatments, which reached significance (ΔΨ_ST_ ≥ -0.2 MPa) only under year x site combinations marked by high evaporative demand. Most notably, in a two-year-long study conducted on Merlot under well-watered conditions in Northern Italy (Herrera et al., 2015), no seasonal effects on Ψ_ST_ were reported for severe shoot trimming made at veraison by leaving only six primary leaves compared to light trimming (i.e., 12 main leaves retained).

Interestingly, when short (~90 cm) and tall (~130 cm) canopies were compared in a semi-arid Tempranillo vineyard along with three irrigation strategies, the tall canopy maintained more negative Ψ_ST_ in both study areas even under full irrigation (Mirás-Avalos et al., 2017). However, it should be noted that, in this specific study, the standard canopy management was represented by the 90-cm-tall canopy, whereas the 130-cm-tall canopy was a purposely extended canopy aimed at creating more limiting conditions.

A typical problem that occurs any time the effect of the management of canopy water use needs to be assessed pertains to determining the time and spatial scales at which the measurements should be taken. Shortening a canopy by severe shoot trimming would indeed cause a perceivable instantaneous response, after which, the progressive compensation in water use would become a primary function of the extent and duration of the lateral regrowth. Additionally, leaving the assessment of whole-canopy transpiration to extrapolation from single-leaf readings has been proven to be quite unsafe Poni et al. (2009a); Medrano et al. (2015). In such a context, Poni et al. (2021) reported a notable use case where the reaction to severe trimming (i.e., six main leaves left) performed at the end of the flowering (end of May) or pea-size (mid-June) stages was followed in terms of season’s whole-canopy transpiration (T_c_) by using an enclosure system (Poni et al., 2014a). Due to the vigorous vegetative regrowth, the earlier trimming already offset the initial T_c_ drop (-23.6% vs. the pre-trimming rates) at the end of July, whereas the later trimming registered an instantaneous T_c_ drop by 44% against the pre-trimming rates while also achieving full T_c_ compensation around the same time.

Overall, the picture emerging from the several studies that attempted to assess shoot trimming as a tool to contain canopy transpiration is quite uncertain. Indeed, two different scenarios can be envisioned. On one hand, in case the technique is applied under non-limiting water conditions, either due to sufficient precipitation or the availability of irrigation, T_C_ is temporarily curtailed and then, progressively replenished by vegetative compensation. On the other hand, if severe shoot trimming is applied under conditions that are not conducive to any significant leaf area compensation, the T_C_ reduction has a more permanent character and is usually reflected in the better leaf water status of the retained leaves.

Within the aforementioned framework, the decision to proceed with severe trimming is driven by the probability of achieving a significant ripening delay with a desirable reduction of the decoupling between sugar and phenolic ripening. In other words, the wish is to slow down the pace of sugar accumulation without affecting flavonoid accumulation. This presents two distinct cases. The first scenario is when severe trimming is performed under conditions that favor partial or full replenishment of the removed leaf area (i.e., early intervention, low crop level, and irrigation availability), thereby originating a dynamic LA/Y evolution after the cut. Here, the existing literature is quite consistent in demonstrating that both sugar and color accumulation are more or less equally delayed not just because of a limiting LA/Y ratio but also due to the direct late-season competition exerted by the lateral shoots (Keller et al., 1999; Poni and Giachino, 2000; Poni et al., 2014b; Santesteban et al., 2017), This practice is quite solid if the goal is to postpone ripening to a cooler period, although the risk is that full ripening is never achieved in climates where the growing season is not that long or when medium-to-late-ripening cultivars are grown.

The second scenario that a fairly late severe shoot trimming might disclose is that, depending on specific conditions (i.e., semi-arid climate, dry farming, and high crop level), the abrupt decrease in the LA/Y ratio is revealed to be almost permanent as negligible vegetative regrowth occurs after the cut. Under such circumstances, two main hypotheses can be drawn. If the post-trimming LA/Y ratio is at a level (i.e., > 1 m^2^/kg) that is considered to be still not too detrimental for ripening, then scant differences in the maturation patterns of untrimmed and trimmed vines are to be expected. Conversely, if severe trimming results in a strong and permanent source limitation (e.g., LA/Y ≤ 0.5 m^2^/kg), then a significant ripening delay should occur. According to (Bobeica et al., 2015; Silva et al., 2017; Zhu et al., 2019), in these circumstances, berries might use a higher proportion of fixed carbon for sugar accumulation under carbon limitation than under carbon sufficiency. Thus, under carbon limitation, the grape berry can manage the metabolic fate of carbon in such a way that sugar accumulation is maintained at the expense of secondary metabolites.

The studies conducted by (Herrera et al., 2015) and (Lu et al., 2023) fall in the former category (i.e., a non-limiting LA/Y ratio even after trimming) and share a mild limitation in berry sugar accumulation, along with reporting no change (Herrera et al., 2015) or minor change (Lu et al., 2023) in the total anthocyanin content. Conversely, (Filippetti et al., 2015; Caccavello et al., 2017; Valentini et al., 2019; Bubola et al., 2022) fall in the other category (i.e., a limiting LA/Y ratio after trimming). Surprisingly, while delayed sugar ripening was registered in all these cases, the flavonoid content, and particularly the total anthocyanin accumulation, was less delayed if not affected at all. This leads to the tempting conclusion that, regardless of the source-to-sink ratio after severe shoot trimming, the vine metabolism tends to delay sugar accumulation more than color accumulation. Despite this being a quite desirable outcome, its physiological bases are unclear. Indeed, two factors can play a role in this. First, if severe shoot trimming is performed late in the season and not followed by any significant canopy regrowth, the obvious instantaneous variation in the amount of the LA/Y ratio is unlikely to be exhaustive. The issue is that the quality of the source progressively deteriorates due to basal leaf aging (Poni et al., 2009b), and this phenomenon might have a stronger impact on sugar accumulation. Second, the total anthocyanin accumulation and degradation are also functions of the local microclimate at the cluster level (Mabrouk and Sinoquet, 1998; Haselgrove et al., 2000). As regards red cultivars, it has been found that berry temperatures exceeding 35°C might inhibit the synthesis of color while also enhancing its degradation (Mori et al., 2007), with some varieties such as Pinot noir, Barbera and Sangiovese being especially sensitive to this (Poni et al., 2018). The cluster microclimate is not directly impacted by late severe shoot trimming, which might justify the lower sensitivity of flavonoid synthesis to the altered LA/Y ratio.

In more general terms, the potential of late and severe shoot trimming to delay ripening has been validated from the aforementioned work. However, the efficacy and transferability of a new practice depend upon several factors, among which practical feasibility and the degree of mechanization are significant. Most of the experiments conducted have imposed manual severe shoot trimming, which leaves six-to-eight main leaves on the main shoot. In a VSP trellis, its mechanical execution is quite impractical regardless of when this operation will be performed, as the commonly used cutter bar machines can perform topping and hedging along an unhindered path only (**Figure 3B**). Therefore, the most severe affordable mechanical cut is the one that is performed when most of the shoots outgrow the top foliage wire. However, this prevents the performance of a truly severe cut as in a canopy wall usually extending for at least 1.2–1.4 m above the main wire, the minimum number of leaves left cannot be lower than 14–16. The alternative is double: to wait until the main shoots have started to bend, so that, after trimming, the number of source leaves removed will be higher. However, waiting until that late for the first trimming can lead to heavy interrow hindrances and difficulties in machinery transit. Second option could be using an over-row rotating disks machine able to negotiate rigid obstacles during the row passage. Indeed, this machine type is slower, more expensive and less flexible than a cutter bar machine. The problem is fully solved when a sprawl canopy is adopted. The free space around the support wire allows any pruning machine to get close to the cordon and perform a short cut (**Figure 3C**) (Poni et al., 2014b).

## 4 Leaf removal

**Figure 4:**
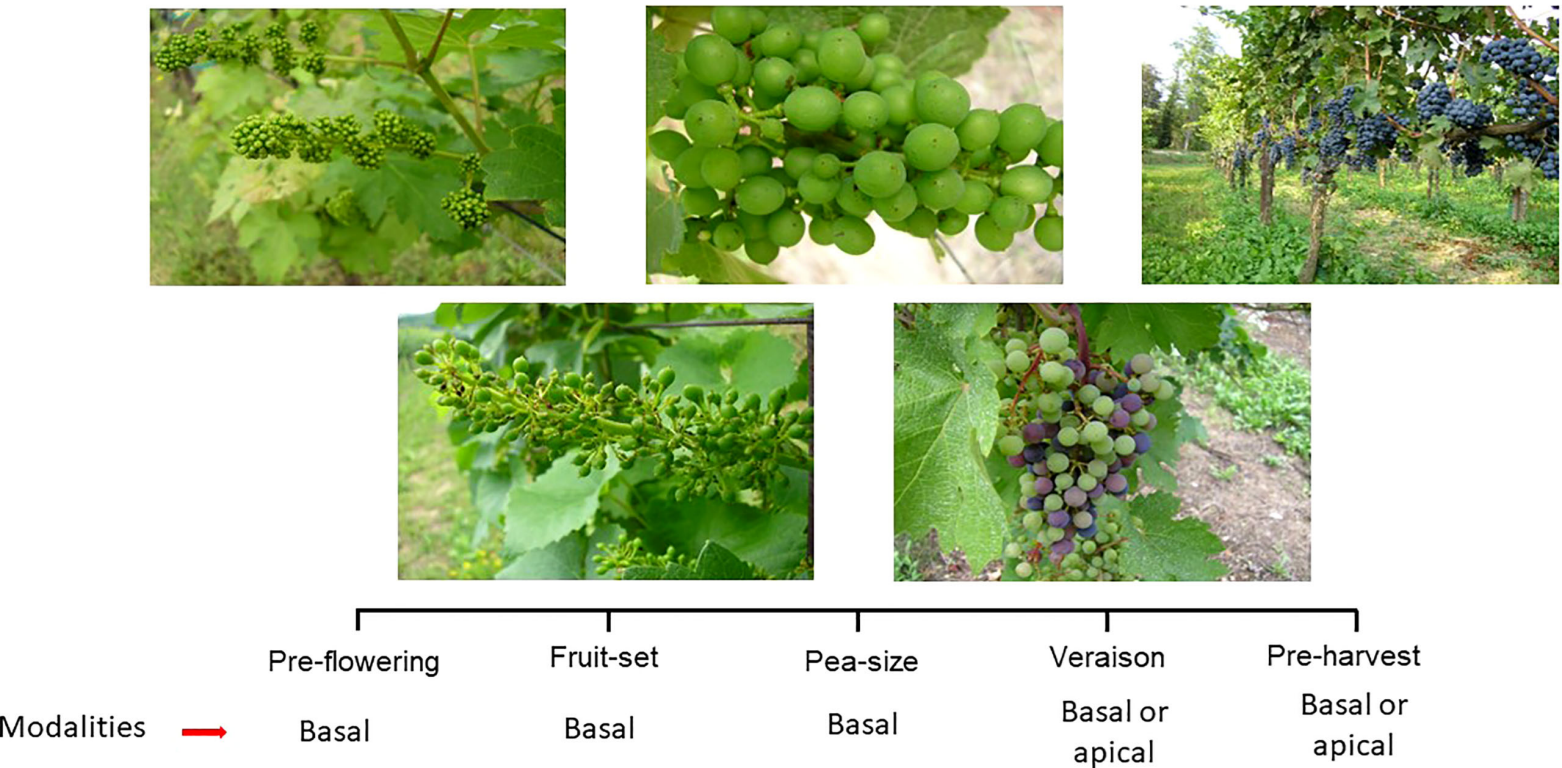
Phenological stages at which leaf removal can be performed on the grapevine. Depending of growing stage and purpose, leaf removal can target different canopy portions (i.e. basal vs apical).

Among all summer pruning operations, leaf removal is the one that has attracted the most attention from the scientific community as well as the one whose modalities and scope have been most significantly affected by global warming. This change has also led to leaf removal getting a different meaning assigned compared to the past. Traditionally, leaf removal has been associated with the plucking of leaves around clusters, which is performed anywhere between fruit set and veraison, to improve light exposure as well as ventilation and facilitate sprays penetration (Koblet et al., 1994; Zoecklein et al., 1998; Kok, 2016; Bubola et al., 2017; Reynolds, 2022). Such goals are still prominent in cool and wet growing regions where the impact of climate change is less and where fruit and wine quality still benefit from lower canopy density and better air circulation at the cluster level (Smith et al., 1988; Zhuang et al., 2014; Frioni et al., 2017). In warmer areas, the current interpretation of leaf removal incorporates two main changes. There is a shared perception that if global warming poses increasing concerns about overheating, sunburn, and desiccation events, leaf removal has to be either applied more judiciously or simply rendered unnecessary. Moreover, leaf removal has been demonstrated to be an extraordinarily flexible operation, as adjusting the timing and severity of the intervention is possible to modulate the effects, thereby piloting them toward a desired direction (i.e., enhancing or delaying ripening) (**Figure 4**).

As regards the actual need for leaf removal, if worries related to multiple summer stresses are increasing (Palliotti and Poni, 2015), then maintaining some leaf cover around the clusters is at least advisable. Recent work on the relationship between the timing of leaf removal and the susceptibility to sunburn (Diago et al., 2012; Ferrari et al., 2017; Gambetta et al., 2019; Gambetta et al., 2021; Rustioni et al., 2023) concluded that early leaf removal (i.e., at fruit set rather than at veraison) will reduce the incidence of sunburn. Moreover, extensive analytics performed by (Gambetta et al., 2021) for photo-protectant compounds (i.e., flavonoids, carotenoids, and chlorophylls) demonstrated that all molecules are especially receptive to the light stimulus during the green phase of berry growth. In fact, their concentration significantly increased after fruit-set leaf removal, whereas a much weaker response was found for late-season leaf removal. From veraison onward, berries lose most of their potential to synthetize photo-protectant compounds; therefore, their acclimation potential is reduced, and their sensitivity to berry sunburn is increased. Furthermore, in cultivars such as Pinot noir, Gamay, Merlot, Chasselas, and Doral (Verdenal et al., 2019), early leaf removal almost doubled the berry skin thickness, whereas in a trial on Barbera (Poni and Bernizzoni, 2010), the same treatment achieved higher relative skin growth than the non-defoliated vines despite having larger berries.

In a paradox, the need of maintaining some leaf cover around clusters during summer has shifted attention to mechanized leaf removal. As a matter of fact, the “imperfect” work of a de-leafing machine—in which some leaves are stripped, others are partially broken, and the rest remain untouched—naturally meets the need of avoiding cluster overexposure, as some leaf cover is always preserved. The evolution of leaf removal has generated two variants of the technique, which have drawn the attention of several research groups.

**Figure 5:**
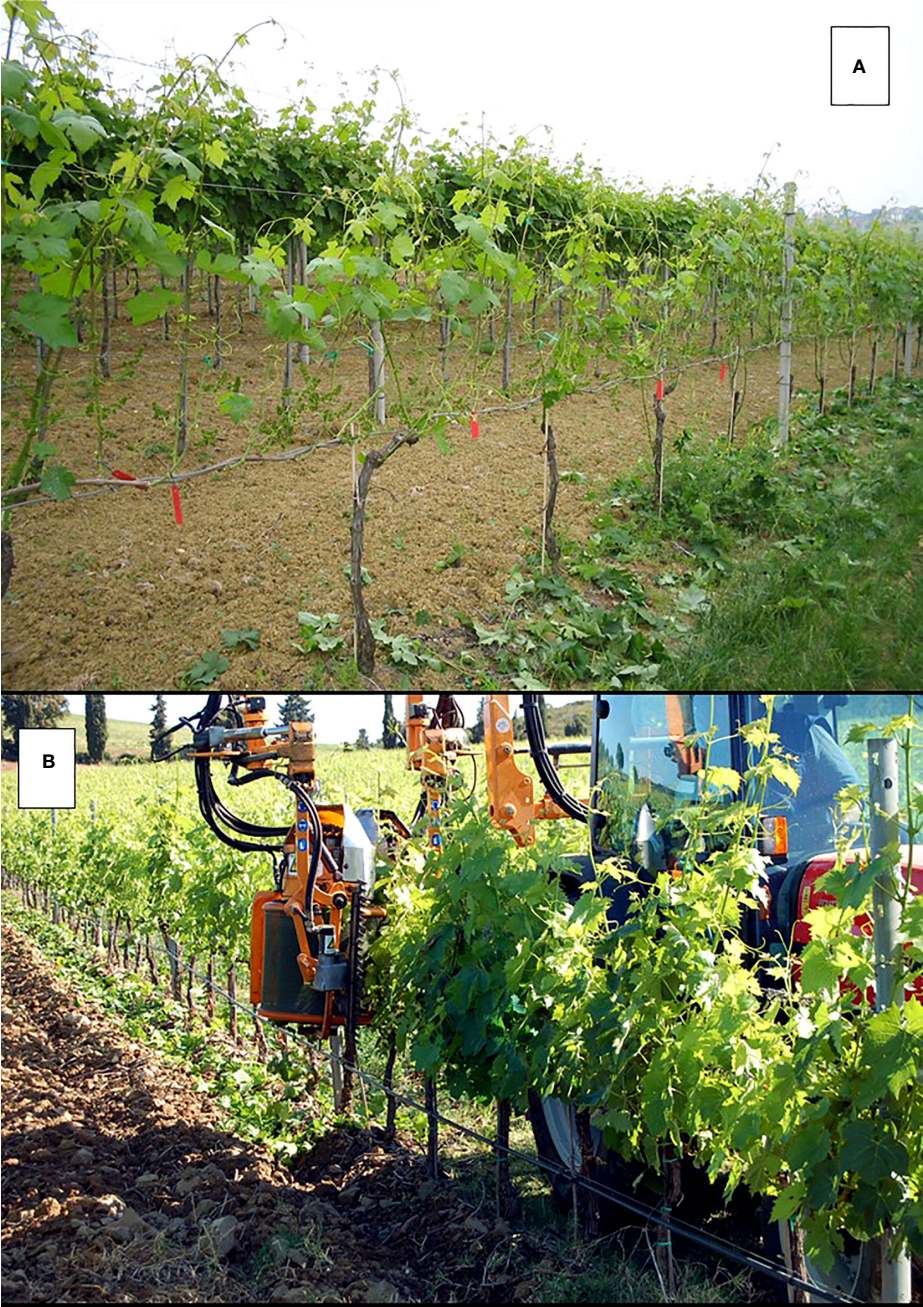
In **(A)** a row section of a vertical shoot positioning (VSP) trellis where manual early (pre-flowering) leaf removal has been completed. The first six basal leaves have been taken out from all shoots under the main purpose to decrease fruit set, control yield and obtain looser clusters less susceptible to rot. In **(B)** the same operation performed at the same stage with a Pellenc™ leaf plucker.

The most tested technique is early (pre-flowering) leaf removal (ELR), the objectives of which are quite different from those of traditional leaf removal (Poni et al., 2006) (**Figure 5**). Based on the strong physiological principle according to which any source limitation caused around the flowering stage will negatively impact fruit set (Coombe, 1962), the technique is considered best suited to cases of high-yielding vineyards with heavy and compact clusters that are quite susceptible to rot and often incapable to reach full ripening. A meta-analysis study was conducted by (VanderWeide et al., 2021), which, after initially identifying 175 publications on the topic of “early leaf removal,” thinned them down to 59 after eight data-curation steps. It returned a clear and consistent picture: ELR systematically lowered cluster rot disease through reduced compactness of clusters, which was mostly due to lower fruit set. Moreover, ELR promoted a significant increase in fruit total soluble solids, which was related to the increase in the LA/Y ratio. Additionally, the total anthocyanin content also tended to increase in ELR, albeit with more variability among the observed responses. Interestingly, the ELR effects were mildly affected by the climate, which supports the hypothesis that the technique has a solid physiological foundation, whereas, cultivar (Verdenal et al., 2019; Mucalo et al., 2021) and rootstock were found to have a larger influence on the success of ELR.

The large-scale adoption of ELR in several viticulture areas around the world and especially where cluster rot diseases are a concern is also due to at least two more factors. First, it has been shown that the operation can be fully mechanized (Intrieri et al., 2008; Tardaguila et al., 2010; VanderWeide et al., 2018) using, at the proper timing (i.e., still no flowers open on the cluster), a mechanical leaf-blower machine that allows for handling larger surfaces (**Figure 5B**). Second, in case no hand labor or machines are available to perform the operation, a viable alternative is using antitranspirants that can coat the leaves and reproduce a source-limiting effect (Intrieri et al., 2013; Mphande et al., 2023). This last solution vastly broadens applicability of this practice as the source limitation is achieved without a physical alteration of the cluster microclimate, which might be quite helpful when the application site features high temperature and radiation loads.

An agreeable side effect of ELR is that the induced yield limitation is proportional to the number of source leaves removed. Some authors (Gatti et al., 2012; Verdenal et al., 2019; VanderWeide et al., 2020; Chalfant and Dami, 2021) have suggested that yield regulation can be effectively achieved through ELR while avoiding collateral negative effects, which might be associated with a cluster-thinning approach where the retained clusters grow more, leading to higher compactness and larger berries. As regards Chambourcin grapes, (Chalfant and Dami, 2021) also reported that performing basal leaf removal at pre-bloom or bloom reduced bud cold injury during the dormant season.

Recently, (O’Brien et al., 2021) demonstrated an original approach to leaf removal where this practice was tested in a sprawl canopy type as a tool to delay ripening in Cabernet Sauvignon without achieving consistent results. However, the source-sink balance of the de-leafed treatments was always above the 1.5 m^2^/kg threshold, and this might explain why the imposed defoliations were rather ineffective.

**Figure 6:**
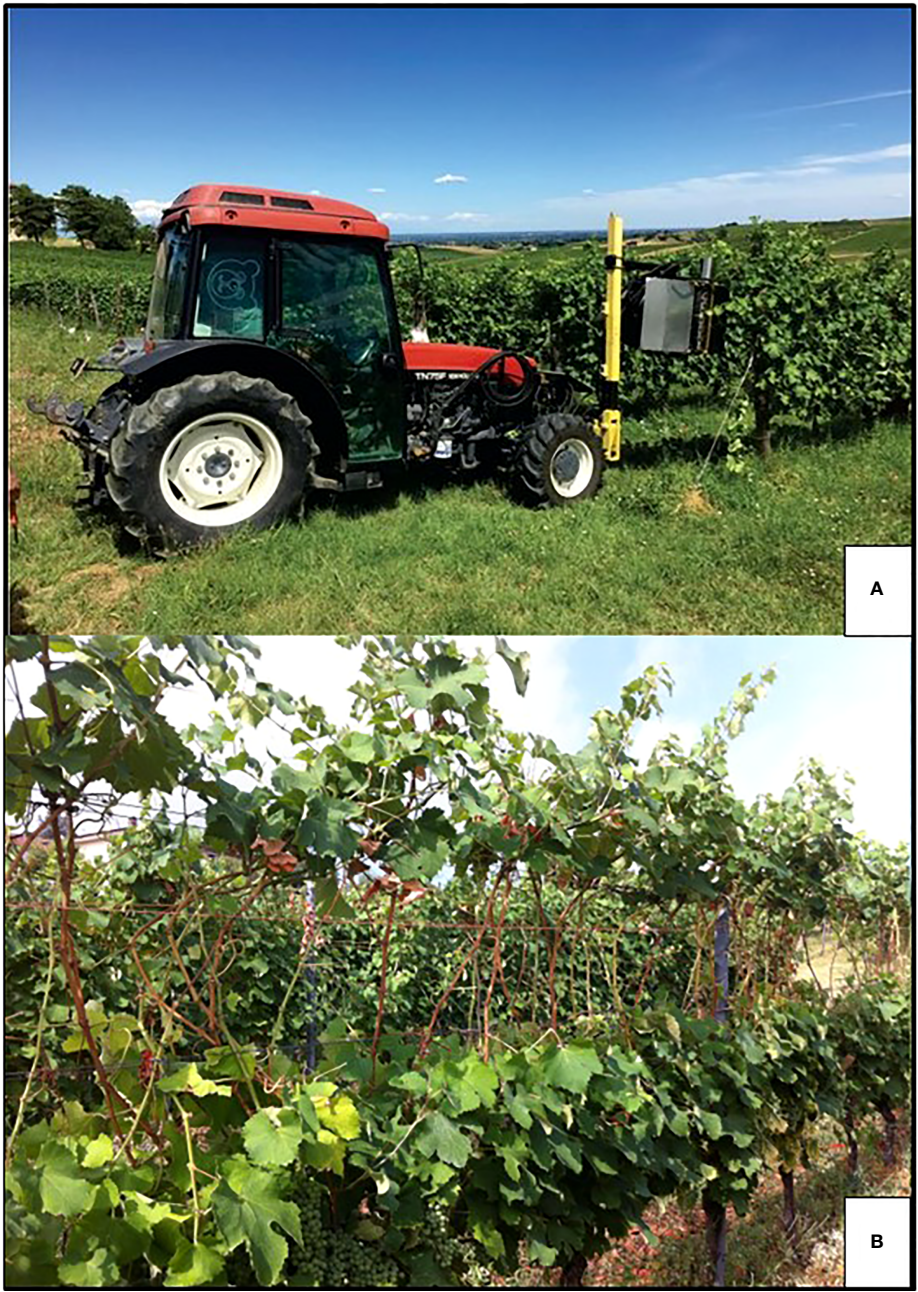
In **(A)**, a leaf plucker machine starts working on the apical canopy portion of a vertically shoot positioning (VSP) trained vine row with the purpose of removing a significant portion of source leaves to induce a ripening delay. In **(B)** the results of the work performed in cv Ortrugo.

The second variant that bursts from traditional leaf removal can be regarded as an up-side-down technique compared to ELR. It is applied late in the season (pre-veraison until a sugar concentration in berries of approximately 10–12°Brix) and targets the apical canopy portions (**Figure 6**) while the cluster microclimate is unchanged (Poni et al., 2013; Buesa et al., 2019; De Bei et al., 2019; Gatti et al., 2019). Overall, the principle that the technique exploits to delay ripening is the same that is pursued in the case of severe trimming, i.e., provoking a calibrated source limitation by removing a portion of functional foliage. However, when compared to severe trimming, one main technical difference exists: apical canopy portion leaf removal aims at opening a window of about half a meter length in a fruitless canopy area. This has two apparent advantages: (i) the operation is fully mechanizable, although two passages per row are needed to optimize leaf removal, and (ii) the task encounters the favor of the driver who knows that defoliation has to be carried out in a fruitless portion, thereby eliminating any fear of possible cluster damage.

The whole-canopy physiology changes brought about by late apical leaf removal have been investigated by (Poni et al., 2013) who studied potted Sangiovese vines to ascertain that both pre- and post-veraison apical leaf removal were effective at significantly delay sugaring and retain higher acidity without affecting phenolic maturity. This happened despite the final source-sink balance of the defoliated treatments was not limiting (LA/Y ratio of approximately 10–11 cm^2^/g and a carbon-to-yield ratio of 9.8–10.4 mg CO_2_/g berry fresh weight), with the latter not differing from the control’s values (11 mg CO_2_/g berry fresh weight). Moreover, a two-year-long field study on Sangiovese mirrored the same results (Palliotti et al., 2013), with vines defoliated at a TSS level of approximately 12°Brix showing, at harvest, 1.2°Brix less than the undefoliated control and an unchanged phenolic maturation. When tested on Semillon and Shiraz in Australia, the technique was found to be ineffective in the first year of application, whereas in the second year, it achieved a delay in ripening of 10 days in Semillon and 20 days in Shiraz, which suggests that the source limitation can be buffered from reserves storage during the first year (De Bei et al., 2019). As shown in (Palliotti et al., 2013) and (Poni et al., 2013), it is remarkable that a sugar ripening delay was obtained even in the absence of a limiting source-to-sink balance. The hypothesis explaining this outcome is that although the final or seasonal LA/Y ratio might not differ between the two treatments, when the leaf removal is performed (around veraison) targeting the youngest apical leaves, the abrupt source decrease is likely strong enough to temporarily limit the sugar trend, which at that time is either at the inflection point or going through the steepest sugar accumulation rate.

## 5 Cluster thinning

By definition, cluster thinning is configured as an extraordinary operation of canopy management that intervenes either when an overcropping status exists or when the sought wine target embraces overripe flavors. If so, a climate change scenario that inherently speeds up ripening should lead to a more conservative application of cluster thinning.

However, it is quite difficult to trace the evolution in the popularity of cluster thinning since a broad meta-analysis study has not yet been conducted. Indeed, in premium areas for red wine, the habit to use cluster thinning as the primary crop load controlling tool (rather than winter pruning) is still widespread. However, despite several publications available on the matter, a primary question related to cluster thinning remains unanswered: is cluster thinning always followed by an increase in grape quality that might offset yield loss and added costs due to the required human labor?

**Figure 7:**
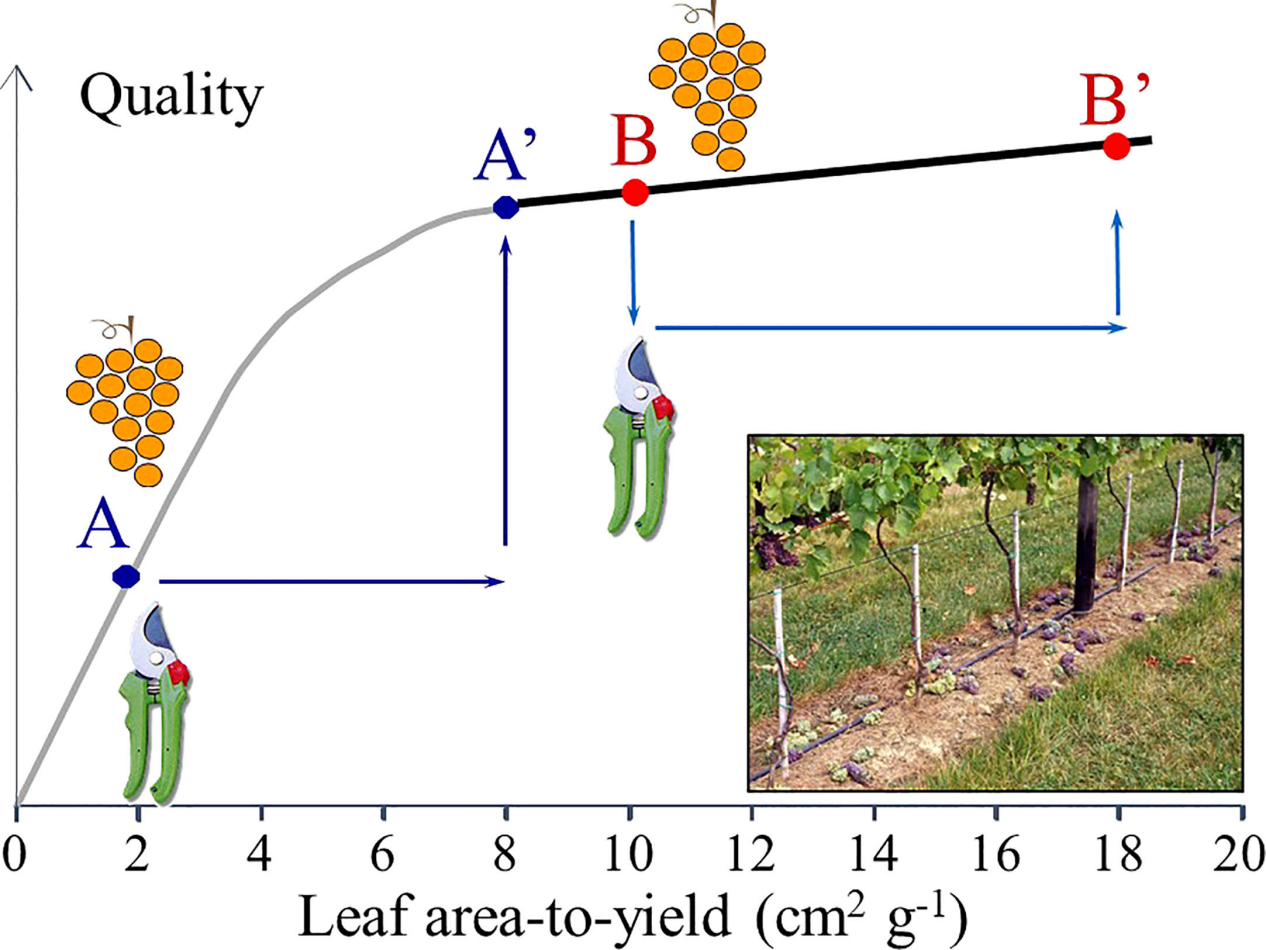
Representation of a physiological hypothesis for prediction of cluster thinning effects. If grape “quality” (defined in general terms as the desired grape composition needed for a given wine style) is plotted vs the leaf area-to-yield ratio, two different cases are envisaged. The gray line represents the improvement in fruit quality from A to A’ when cluster thinning is done in over cropped vines. The black line represents the negligible effect of cluster thinning in fruit quality from B to B’ when vine source-sink is properly balanced.

The following physiological principle (**Figure 7**) should drive the cluster thinning operation: if the anomalous values in the LA/Y ratio (e.g., lower than 1 m^2^/kg) or the Ravaz index (e.g., higher than 8–10 kg/kg) warn about a likely overcropping status, then cluster thinning should lead to the expected results of significantly improving grape and wine quality while helping to remain within yield limits imposed by law. However, when cluster thinning is performed in vines with adequate crop load, the advantages of these technique significantly diminish, resulting in reduced economic return due to the additional cost required to implement the technique (Berkey et al., 2011; Preszler et al., 2013; Schamel and Schubert, 2016).

Summary of main results of 20 papers on cluster thinning are shown in **Table 1** which also reports, along with the effects on yield and grape quality, the evaluation of the LA/Y ratio or the Ravaz index (when available) in the different treatments. An overall analysis of the group of 20 papers led to the following conclusions:

- The aforementioned hypothesis holds true in several cases (Keller et al., 2005; Intrigliolo and Castel, 2011; Sun et al., 2012; Zhuang et al., 2014; Mawdsley et al., 2019; Martínez-Lüscher and Kurtural, 2021; Copp and Levin, 2022), although significant deviations were also found (Gatti et al., 2012; Sivilotti et al., 2020; Aru et al., 2022 a, b).
- In most cases cluster thinning resulted in a significant final yield reduction, suggesting that yield compensation mechanisms from the retained clusters have been overall minor. Exceptions were data from Intrigliolo and Castel (2011) and Mucalo et al. (2022).
- In the few studies where the same CT treatments were repeated for three years on the same vines, the adjustment to CT increased over time to offset between-treatment differences in the second or third year (Mawdsley et al., 2019; Martínez-Lüscher and Kurtural, 2021; Netzer et al., 2022).
- In almost all the studies where CT induced significant yield limitation, the total soluble solids at harvest were the most responsive variable (Reynolds, 1989; Dami et al., 2006; Gil-Muñoz et al., 2009; Kok, 2011; Reščič et al., 2015), whereas the total anthocyanin content was often less affected (Guidoni et al., 2002; Santesteban et al., 2011; Sun et al., 2012; Copp and Levin, 2022).
- It was apparent that the effects induced by cluster thinning were sensitive to cultivar and specific environmental conditions of the site, rendering the forecasting of its effects hard to predict and endangering the repeatability of the effects.

**Table 1:** Synoptic information from 20 research papers on the impact of cluster thinning strategies on yield, fruit quality and source to sink balance of grapevine.

Country/ cultivar	Trial years	Timing/ intensity of CT	Effects on yield	Effects on grape quality	LA/Y ratio or Ravaz index	Notes	Reference
USA/Cabernet Sauvignon	3	Fruit set/33-66% fruit removal	–	+TSS	++ LA/Y	3^rd^ Year, TSS and LA/F ns	(Martínez-Lüscher and Kurtural, 2021)
Italy/Refosco dal peduncolo rosso	2	Pre-veraison/50% fruit removal	–	+TSS, + color	++ LA/Y	LA/Y in C = 1.90m^2^/kg; LA/Y in CT = 3.34m^2^/kg	(Sivilotti et al., 2020)
USA/Pinot noir	3	4,8,12 weeks after bloom	-(Y1); ns (Y2-3)	+ or ns TSS;	Ravaz, ns	Ravaz varying between 1-3 kg/kg	(Mawdsley et al., 2019)
Australia/Semillon - Shiraz	2	Veraison/50% fruit removal	–	++TSS	N/A		(Wang et al., 2019)
USA/Pinot noir	3	Peppercorn/1 cluster/shoot center	-or –	+ or ns TSS; ns color	-or ns Ravaz		(Copp and Levin, 2022)
Denmark/Solaris	1	Veraison/66% fruit removal	–	++TSS	ns, LA/Y	LA/Y in C = 13 m^2^/g; LA/Y in CT = 10 cm^2^/g	(Aru et al., 2022a; Aru et al., 2022b)
Israel/Malbec	3	Pre-veraison/50% fruit removal	-(Y1); -ns (Y2-3)	+ (Y2) or ns (Y1 and 3)	N/A	C and CT wines perceived as similar	(Netzer et al., 2022)
Croatia/Maraština	1	Veraison/35% fruit removal	ns	ns	N/A	CT wines had better aroma than C wine	(Mucalo et al., 2022)
USA/Chambourcin	5	10(L), 20(M), 30(H) clusters/vine	M vs H ns - in L in 3Y out of 5	+TSS in L in 3Y out of 5	N/A		(Dami et al., 2006)
Spain/Tempranillo	2	11(L), 20(M), 27(H) clusters/vine	Occasional lower yield in L	Occasional higher TSS in L	LA/Y varying from 0.7 to 3.1 m^2^/kg	Increased response to thinning for LA/Y < 1.5 m^2^/kg	(Intrigliolo and Castel, 2011)
Turkey/Sauvignon blanc	1	4,6,8,10,12 weeks after bloom/1 cluster per shoot	-35% vs C	+TSS	N/A		(Kok, 2011)
Slovenia/Blauer Portugieser	3	Pea size/25 and 45% fruit removal	-or ns in25% – in 45%	ns TSS in 25% +TSS in 45%	N/A	25% fruit removal quite ineffective	(Reščič et al., 2015)
USA/Cabernet Franc	2	Fruit set, 3 weeks before veraison and veraison/40(L) and 80(H) clusters/vine	-38% in the L treatment	ns TSS + color in L for second year only	Lower Ravaz in L in both years		(Zhuang et al., 2014)
Italy/Sangiovese	3	Flowering and lag phase; 50% fruit removal	-45% vs C	++ TSS ++ color	LA/Y = 1.03 m^2^/kg in C; LA/Y = 1.65 m^2^/kg in CT		(Gatti et al., 2012)
USA/Coron	2	Pea size/removal of distal clusters in CT	– in CT	+ TSS ns color	Ravaz varying from 2.3 and 7.1 kg/kg	Very vigorous vines	(Sun et al., 2012)
USA/Cabernet Sauvignon, Riesling, Chenin blanc	5	Pea size and pre-veraison/30% to 39% fruit removal	-17% to 36% in CT	ns TSS ns color	LA/Y = 1.7-1.9m^2^/kg in C; LA/Y = 1.6-3.7 m^2^/kg in CT		(Keller et al., 2005)
Spain/Tempranillo	4	Veraison/one cluster/shoot in CT	- in 10 out of 12 vineyards	+ TSS in 6 out 12 vineyards; + color in 4 out of 12 vineyards	N/A		(Santesteban et al., 2011)
\Italy/Nebbiolo	3	Pea size/50% fruit removal	-21% in CT	+TSS and color in 2 out of 3 years	N/A		(Guidoni et al., 2002)
Spain/Tempranillo and Shiraz	3	Pre-veraison/one cluster/shoot in CT	-40% in CT	+TSS +color	N/A		(Gil-Muñoz et al., 2018)
Canada/Riesling	3	Fruit set/1 and 2 clusters/shoot vs C	-30% in one cluster/shoot vs C	++TSS	N/A	No differences in final wines	(Reynolds, 1989)

Y, year; C, control; CT, cluster thinning; L, low; M, medium; H, high; LA/Y, leaf area-to-yield ratio in m^2^/kg or cm^2^/g; Ravaz index as yield-to-pruning weight ratio (kg/kg); TSS, total soluble solids; color = total anthocyanins unless otherwise stated. One single – or + sign means significant vs C at p < 0.05. Two – or + signs mean significant vs C at p < 0.01. ns = non-significant.

Global warming is causing excessively fast ripening, especially in semi-arid areas with increasing decoupling between sugar accumulation and phenolic ripening (Nicholas, 2015). Among the several solutions available to slow and postpone ripening to a cooler period (Palliotti et al., 2014), the reuse of unripen, thinned clusters was also evaluated (Kontoudakis et al., 2011) with the specific aim of reducing alcohol content and pH. The idea was to use a fraction of the thinned clusters at veraison to produce a very acidic must (TSS at 5° Brix, total acids at 17.8 g/L, and pH = 2.78) in Merlot, Cabernet Sauvignon, and Bobal. At the end of fermentation, the wine was treated with activated carbon and bentonite to remove phenolics and aggressive green flavors and obtain an odorless as well as colorless product. To adjust the composition of wines derived from the normal harvests, an aliquot of the green must was added to replace an equivalent amount of standard must for each cultivar. Within batches of 8 kg of grapes and a must yield of 6.4 L, the replaced must fraction was 0.85, 1.50, and 2.0 L for Cabernet Sauvignon, Merlot, and Bobal, respectively. The results met the expectations, as the final alcohol content in the blended wines was reduced by 0.9% (Cabernet Sauvignon), 1.7% (Merlot), and 3.0% (Bobal) compared to traditional wines. Moreover, due to a significant reduction in wine pH, blended wines also registered higher total anthocyanin concentrations.

## 6 Summer pruning and interactions with late frost and hail events

A very consistent trait accompanying the climate change era is, indeed, the increase in both frequency and severity of so-called “extreme” weather events (AghaKouchak et al., 2020). Climate becomes extreme when damaging events turn unusually violent or anomalous in terms of their frequency and duration. In Europe, it has been estimated that the frequency of extreme events registered over the last three decades causing significant economic losses has increased by about 60% (Sánchez et al., 2004; Beniston et al., 2007). While we have covered the relationship between specific summer pruning operations and drought and/or sunburn in the single chapters of this review, late frost and hail occurrences require a specific treatment.

Within a global warming scenario, two effects in particular can contribute to render late frost a fearsome event: i) earlier phenology is one of the most characteristic and consistent changes induced by higher heat summations. In the grapevine, a more advanced bud burst can overwhelm any benefit derived from global warming, leading to a reduction (rather than a widening) of the “frost free period” (Leolini et al., 2018); ii) in the event of a potentially damaging temperature, the vegetative development of the vine can be definitely more advanced, thus inherently increasing damages to tissues (Fuller and Telli, 1999).

A technique named “late winter pruning” (LWP) has been tested mostly over the last two decades as a prevention or mitigation tool against late frost damages in vineyards (Poni et al., 2022).

**Figure 8:**
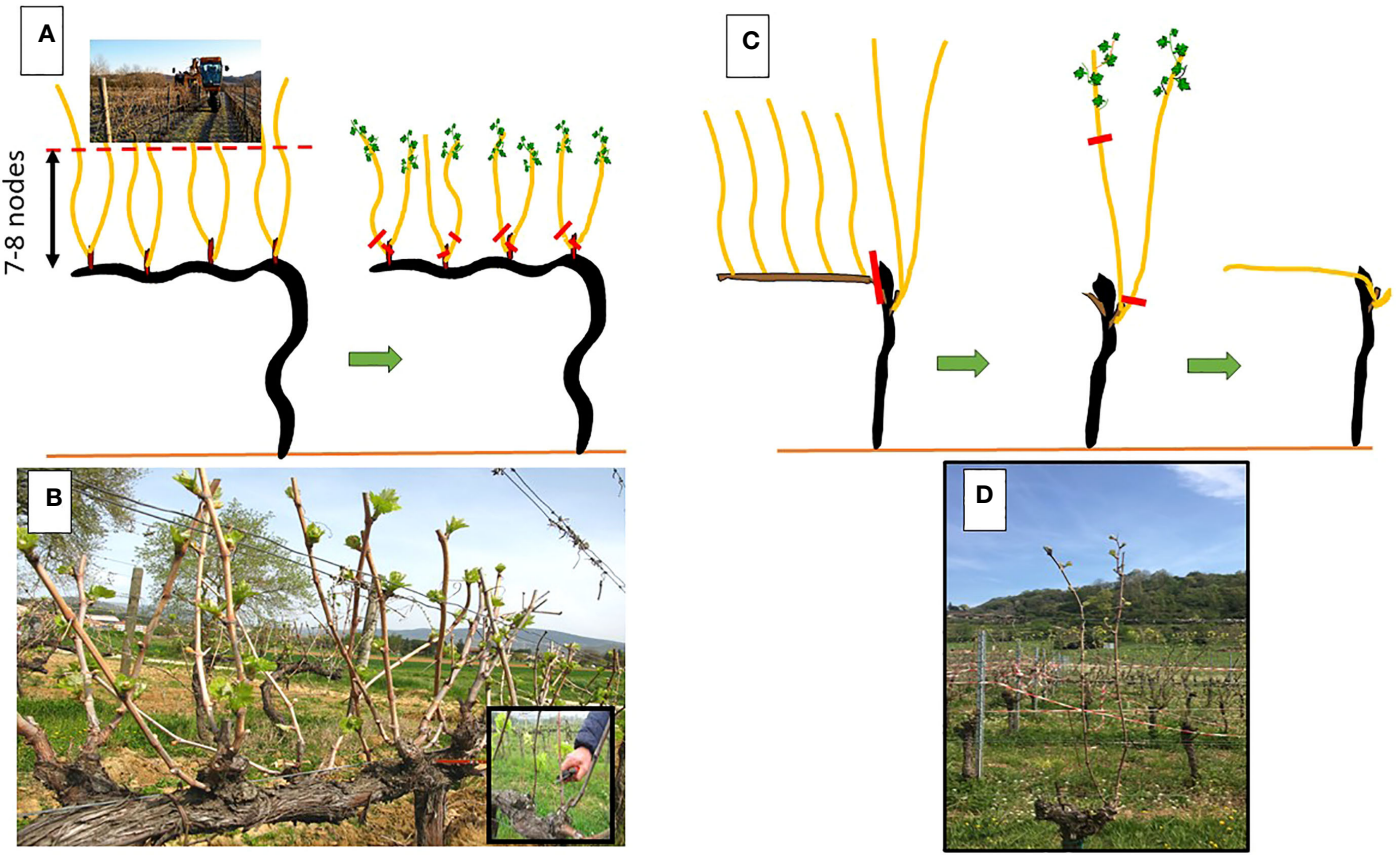
Representation of the Late Winter Pruning (LWP) protocol applied on spur-pruned **(A, B)** and cane pruned **(C, D)** vines. In spur-pruned vines, a mechanical pre-pruning **(A)** is performed during dormancy to shorten the canes **(B)** before final spur pruning is made (inset of panel **B**). In cane pruned vines, cane selection of unshortened canes is made in winter **(C, D)** and then followed by final shortening and positioning of the cane along main wire. Red lines indicate pruning cuts. In both pruning systems, recommendation is not to wait beyond the stage of 2-3 unfolded leaves to perform the late cuts.

This LWP is based on an extraordinarily simple physiological principle that can be related to grapevine acrotony. In the grapevine, regardless of the length and position of the productive unit (i.e., a spur or a cane of variable length), the distal nodes are the first to commence growth in spring, after which the proximal buds gradually follow. Based on this principle and to prevent or minimize late frost damage, the goal of LWP is to postpone final winter pruning until shoot growth has not commenced on the apical nodes. Advantages are: i) time elapsing between date of budburst and actual late winter pruning date is the first mechanism lowering chances to incur into frost damages; ii) lag time needed to the basal nodes to burst and reach a growth stage susceptible to frost (e.g swollen bud onward) is an additional warrant against frost damage. Details of the LWP protocols suited for either spur pruned and cane pruned vines are described in Poni et al. (2022) and also shown in **Figure 8**.

In the above- cited review paper, summary of the results from 21 trials which applied LWP under an array of locations and cultivars leads to the following main conclusions: i) bud burst was delayed by 5 to 56 days according to local condition and experimental layout; ii) yield was either unchanged or variably reduced vs a standard winter pruning treatment. A common trait of several works is that when the final pruning or hand finishing takes place beyond the stage of 2-3 unfolded leaves, yield of the current season can be seriously lowered, and iii) in a significant number of cases the bud burst delay caused by LWP carries on until harvest with notable improvement especially in terms of total anthocyanins and phenolics; iv) an inherent hurdle of this kind of study is that, even if all the treatments are correctly planned, it is virtually impossible to predict the actual occurrence of a significant frost damage. However, such a situation occurred on 29 April 2019 during a two-year trial on Lemberger, when a freezing event occurred when the phenological stage of the control averaged between woolly buds and green leaf tips visible, respectively (Persico et al., 2023). In 2019, late-pruned (1 May) vines had 61% greater yield than control vines, reflecting differences in shoot freeze damage between the two treatments. Moreover, final grape quality was not affected.

A close connection between summer pruning and frost damage also occurs when it has to be decided if and how to intervene to facilitate vine recovery and yield capacity restoration. While actions to be taken need surely to take into account timing and severity of frost damage, here we would like to concentrate on different behaviors to be maintained according to the adopted pruning system.

While in a spur-pruned cordon care should be taken that the post-frost new shoots grow upward along the foliage wires, thereby safeguarding their integrity, in cane-pruned systems, some additional concerns might arise. In a Guyot trellis, after a very severe frost damage, secondary shoots are usually developed at each node position along a horizontal or arched cane. If the specific site allows vigorous regrowth, such shoots can be maintained to build a new efficient leaf area. Conversely, in low-vigor sites or locations suffering, for instance, from summer drought, allowing all the shoots to develop increases the risk that a relatively low growth potential is partitioned among an excessive number of shoots. The final undesirable outcome is that no good renewal canes are borne on the head of the vine. If this is the case, anticipating the removal of the damaged canes can result a winning choice as the residual vine regrowth potential will be concentrated on the few buds that will develop from the vine head.

A trickier situation occurs when the mortality of the primary shoots after the frost event is lower; for instance, < 50% of the total shoots. Once again, different pruning systems will require different approaches. In spur-pruned cordons, chances of achieving a decent crop level as well as canes robust enough to warrant good spur selection for the next cropping are quite high, even in cases where no post-frost operations are executed. Conversely, for long-cane pruning lack of intervention would likely be inappropriate. For instance, laterals already developing from the basal nodes of the partially injured shoots will soon achieve apical dominance and will become the primary vegetative sinks. In a cane-pruned system, those laterals might represent a wasteful process as they usually stem from canopy positions unsuited to provide a renewal cane. Moreover, the energy the grapevine invests into that growth will be detrimental to the development of a long cane in a suitable position (i.e., on the head of the vine and possibly below the supporting wire).

For hail events, forecast models predict a progressive decrease of relative humidity and an increase of convective instability, mostly due to atmospheric overheating; in turn, this might lead to an increase in the frequency of hail events as well as the possibility of larger hail grains (Raupach et al., 2021). As in the case of late frost damages, it would be useful to address advisable post-hail summer pruning interventions. For severe hail damage (i.e., canopy defoliation higher than 50%) occurring at a still early stage (roughly by fruit set), prompt intervention is mandatory to safeguard normal cropping level for the next year. Severe damage is likely to compromise the integrity of the canes that will have been used for the replenishment of fruiting spurs or new canes at winter pruning; therefore, it is crucial to concentrate on the post-hail vine regrowth of those growing points that are deemed useful for winter pruning.

However, the scenario changes again in relation to spur-pruned or cane-pruned systems. In spur-pruned cordons, the main goal is to form new canes with basal portions that are thick and mature enough for the successful selection of future spurs. Conversely, in cane-pruned systems, the new cane must be long enough to fill the space available along the support wire. Probability that the new canes stimulated by the post-hail intervention will complete a regular bud induction and differentiation process depends on the speed of growth resumption as well as the environmental conditions (namely light and temperature) accompanying the growth of the new shoots/canes. If, besides a standard sanitation spray aimed at disinfecting wounds and facilitating the healing process, a “no intervention” option is chosen, the worst possibility is that most of the regrowth vine potential will be concentrated on fostering the regrowth of erratically distributed laterals, which mostly happens at canopy positions unsuited to winter pruning needs.

Consequences of hail damage are especially fatal when it occurs late in the season, e.g., at a time when even the growth of laterals has almost ceased. Under such circumstances, in addition to the damage to the clusters usually being more severe, it becomes very difficult, if not impossible, to promote the growth of new wood for the forthcoming winter pruning. However, in a spur-pruned system, late-season hail damage followed by prompt and aggressive reform pruning with the aid of any possible forcing tool (e.g., late-season irrigation, foliar fertilization, removal of a competitive cover crop, etc.) might still allow the formation of some new wood, which might give rise to a few renewal spurs. But this is very unlikely to happen in a cane-pruned system, and, quite often, a late hail event causes the cropping function to be compromised for two consecutive years, rendering business sustainability a very serious concern.

## 7 Crop forcing in *Vitis vinifera*: a summer pruning consequence?

An increasing number of studies have reported on the shifts in timing and length of the growing season, based on phenological, satellite, and climatological studies (Bradshaw and Holzapfel, 2006; Christiansen et al., 2011; Cook and Vizy, 2012). The evidence points to a lengthening of the growing season of ca. 10–20 days in the last few decades, where an earlier onset of the start is most prominent (Linderholm, 2006). Variations in the timing and length of the growing season may not only have far-reaching consequences for plant and animal ecosystems, but persistent increases in the length of the growing season may lead to long-term increases in carbon storage and changes in vegetation cover, which may affect the climate system.

**Figure 9:**
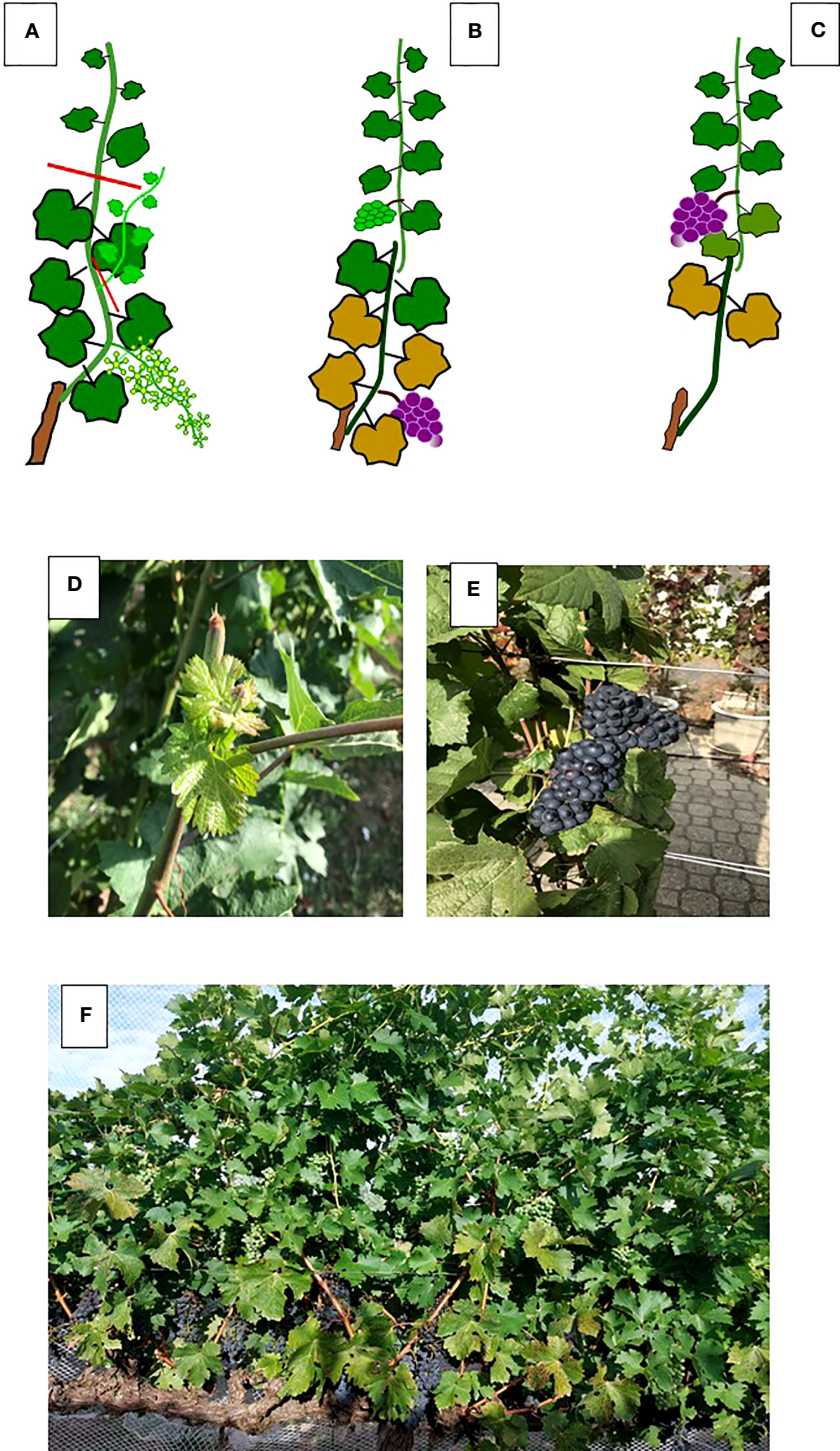
Representaion **(A–C)** of the crop forcing technique leading to two harvests in the same season. In **(A)** main shoot is trimmed at six leaves and all laterals removed. This will unlock the dormant buds. In **(B)**, the primary crop is ripen, whereas the forced crop is at about lag phase of berry growth; in **(C)** the forced crop is ripen while leaf shedding has already started from the basal part of the canopy. In **(D)** a detail of the forced shoot originated by the dormant bud and in **(E)** two forced Pinot noir clusters close to maturity. In **(F)** a Cabernet Saubvignon canopy where, in the lower portion, the primary clusters can be seen whereas, in the upper portion, the still green forced clusters are also visible.

As a deciduous fruit tree, the grapevine is, of course, quite receptive to such changes (Jones and Davis, 2000; Mesterházy et al., 2018), and a lengthening of the growing season is stimulating new agronomic approaches, one of which, unsurprisingly, is based on various combinations of summer pruning. The terminology of “crop forcing” is quite effective in describing an attempt to shift cropping and maturation at a later stage compared to calendar ripening dates. The physiological principle behind the technique is quite simple: in a *Vitis vinifera* L. cultivar grown in a temperate or continental climate where buds typically undergo winter dormancy, the cropping cycle and ripening can be consistently postponed if the dormant bud is forced to grow during its first year of induction and differentiation (**Figure 9**). This happens if its para-dormancy status is broken making the dormant bud acting as a prompt bud. The pioneering study that paved the way for this technique was by (Gu et al., 2012) who, in the hot environment of Fresno, California, trimmed the primary shoots to six nodes at different dates while also removing any main leaf, laterals, and primary clusters. When the trimming was performed by pea-size (at the latest), the results were astonishingly good. The forced (F) crop was only 13% less than the primary crop achieved on the unforced control (C) vines (6.45 kg/vine vs. 7.35 kg/vine). The forcing shifted the harvest by over two months (from mid-August to mid-October and even further) and the fruits from the forced crop had similar TSS, smaller berries, lower pH levels, higher acidity, and enhanced total anthocyanin and phenolic concentrations compared to C vines.

Since then, other studies have followed. However, these are difficult to compare as a “crop forcing” technique might follow different strategies, which depend upon the type of organs that are retained on the vine at the time when the forcing is imposed. In their study, Gu et al. (2012) left no organs on the trimmed shoots of the forced vines. The same approach was followed in more recent studies (Martínez et al., 2019; Martínez-Moreno et al., 2019; de Toda, 2021; Martinez De Toda, 2021; Cabral et al., 2023; Lavado et al., 2023a; Lavado et al., 2023b) conducted on Touriga Nacional, Tempranillo, and Maturana tinta, which delivered a surprisingly consistent outcome: in all the studies, the forced crop (replacing in full the removed primary crop under the current circumstances) had similar TSS to the unforced control while displaying distinctly higher acidity (especially malic), lower pH, and greatly enhanced total anthocyanin and phenolic concentrations. The weak point shared by all the studies is that the grape yield borne on the forced shoot was severely curtailed compared to the primary crop harvested on the unforced vines, as the yield reduction was 70–85% in Tempranillo and Maturana tinta (Martínez et al., 2019), 57% in Tempranillo (Lavado et al 2023a), 88–90% in Touriga Nacional (Cabral et al., 2023). Evidence suggests that such a negative impact on yield carried by the forced primary shoots is largely due to the severe source limitation, which parallels the unlocking and development of the primary bud whose differentiation might result incomplete originating clusters with fewer and smaller berries. Moreover, depending on the timing at which the forcing is performed, the dormant bud might not have yet completed the floral induction process, thereby resulting in several vegetative shoots. Such yield constraint associated with the high amount of labor required for organ removal renders this practice economically unsustainable.

A new light has been shed on the forcing technique by authors who followed a different approach. As already discussed by (Gu et al., 2012), crop forcing application can differentiate depending on the types of organs that are retained or removed from the vine. Therefore, (Poni et al., 2020; Martinez De Toda, 2021; Poni et al., 2021) decided to retain primary leaves and clusters while removing laterals after severe trimming of the primary shoots. The purpose behind this was to head toward “double cropping” with an early and late harvest to be completed on the same vines (**Figure 9**). The same forcing type was preliminarily tested by (Gu et al., 2012), who reported a 74% yield decrease for the forced shoots compared to the primary crop setting at 6.85 kg/vine. More recently, “double cropping” was evaluated on a two-year basis in Grenache, Tempranillo, and Maturana tinta by (Martinez De Toda, 2021) and in Pinot noir (Poni et al., 2021) following a very similar experimental protocol, where the main shoots were trimmed to six-to-seven nodes between the end of the flowering and the pea size stage, and all the laterals were progressively removed. In the former experiment, across cultivars, the time distance between primary and forced harvests went from 32 to 52 days (the latest harvest was on November 4 in Tempranillo) and TSS was lower in any forced treatment, whereas the acids and the total anthocyanin concentration were greatly enhanced. Overall, the forced crop per vine averaged over the three cultivars was 1.24 kg, which is 42% of the primary crop. The results obtained on Pinot noir were similar, if not better. The crop obtained in the forced primary shoots was about 40–50% of that borne on the regular primary shoots, and, interestingly, the second crop’s grape quality scored higher TSS, total anthocyanin and phenolic concentrations, and total acidity. While ripening in the standard crop was reached within the second week of August (which is a rather usual pattern for an early ripening variety grown in an environment assuring 1800–1900 GDD), the forced crop was harvested on October 7 and 8, respectively.

An enticing feature of this daring technique is not only its ability to pursue two harvests within the same season but also its potential to furnish batches of grapes of such different compositions as to allow different market segments to be profitably targeted. In terms of acceptance from growers, the “double cropping” version has an inherent advantage: it is difficult to convince any grower to remove the entire primary crop under the “hope” of obtaining a forced one. At present, more work is required from an operational standpoint to ease the quite laborious shoot trimming and lateral removal operations. Technically, shoot trimming already is an easy and fully mechanized summer pruning practice in vineyards; however, under the protocol envisaged by bud forcing, the trimming should take place above six-to-eight nodes on the main shoots, which means that the machine will not act without the hindrance of posts, stakes, and wires. A solution to this problem is provided by replacing a traditional cutter bar trimmer (which performs topping on vertically positioned shoots when they have outgrown the top foliage wires) with an over-the-row rotating disk machine. The principle of functioning for such machines allows for the navigation of posts and stakes, and once the distance of the bottom pair of disks from the height of the cordon or cane is regulated to correspond to an average cut at six-to-eight nodes, the machine action could also be revealed to be effective at removing or stripping all the young laterals.

In summary, the crop forcing technique applied in a double cropping mode is very interesting. However, to complete a simultaneous double reproductive cycle and active vegetation having to be sustained in mid-summer, vines need high vigor and high resources in terms of nutrients and water. As a matter of fact, Martinez De Toda, (2021) heavily irrigated vines with 4.5 L per day, whereas Poni et al. (2021) used potted grapevines. While longer term studies are needed, initial recommendation would be to start trialing this practice in irrigated vineyards and/or environments having no major summer drought occurrences.

## 8 Conclusions

The modalities and scope of vineyard summer pruning are evolving along with progressive effects bound to global warming. Although generalization is a difficult task, the following main changes seem to stand out:

1) An almost globally felt requirement is that more leaf cover around the clusters is needed today compared to the past. Such a need would render operations such as shoot thinning and leaf removal less stringent, as these exert more of a direct impact on fruit microclimate than others. Moreover, the same need should give more impulse to mechanical leaf removal compared to hand leaf removal.
2) Summer pruning represents a burden in terms of workload. Practices, such as shoot trimming, trunk de-suckering, and leaf removal, are easily mechanizable, whereas others, such as shoot and cluster thinning, are still bound to laborious hand work, which has an impact on vineyard profitability.
3) The most prominent advancement has been a change in mentality, which has configured a given summer pruning as not just a “necessary evil” but rather as a tool to pilot ripening into a desired direction. Early basal leaf removal, apical to the cluster leaf removal, and, more recently, the crop forcing that eventually leads to two harvests in the same season, are very good working examples of this.
4) Summer pruning will likely be impacted by precision viticulture and robotics, and first attempts to automize highly selective operations such as shoot and cluster thinning are underway.

## Author contributions

All authors listed have made a substantial, direct, and intellectual contribution to the work and approved it for publication.
